# Molecular Characteristics and Pathogenicity of *Porcine Epidemic Diarrhea Virus* Isolated in Some Areas of China in 2015–2018

**DOI:** 10.3389/fvets.2020.607662

**Published:** 2020-12-07

**Authors:** Linyang Yu, Yanling Liu, Shuangyun Wang, Leyi Zhang, Pengshuai Liang, Lei Wang, Jianguo Dong, Changxu Song

**Affiliations:** ^1^College of Animal Science & National Engineering Center for Swine Breeding Industry, South China Agriculture University, Guangzhou, China; ^2^School of Animal Husbandry and Medical Engineering, Xinyang Agriculture and Forestry University, Xinyang, China

**Keywords:** PEDV, pig, phylogenetic analysis, S gene, pathogenicity

## Abstract

Since 2010, *Porcine epidemic diarrhea virus* (PEDV) has caused severe diarrhea disease in piglets in China, resulting in large economic losses. To understand the genetic characteristics of the PEDV strains that circulated in some provinces of China between 2015 and 2018, 375 samples of feces and small intestine were collected from pigs and tested. One hundred seventy-seven samples tested positive and the PEDV-positive rate was 47.20%. A phylogenetic tree analysis based on the entire S gene showed that these strains clustered into four subgroups, GI-a, GI-b, GII-a, and GII-b, and that the GII-b strains have become dominant in recent years. Compared with previous strains, these strains have multiple variations in the SP and S1-NTD domains and in the neutralizing epitopes of the S protein. We also successfully isolated and identified a new virulent GII-b strain, GDgh16, which is well-adapted to Vero cells and caused a high mortality rate in piglets in challenge experiments. Our study clarifies the genetic characteristics of the prevalent PEDV strains in parts of China, and suggests that the development of effective novel vaccines is both necessary and urgent.

## Introduction

*Porcine epidemic diarrhea virus* (PEDV) is the etiological agent of porcine epidemic diarrhea (PED), a severe diarrhea disease in piglets that is characterized by severe watery diarrhea, vomiting, dehydration, weight loss, and nearly 100% mortality (1). PED occurred sporadically around the world in 1990–2009, but in 2010, an acute and severe outbreak of PED in piglets occurred in China and spread to other Asian countries, causing large economic losses (2–8). In April 2013, PED suddenly erupted in the United States, causing many piglets to die, and the mortality rate in suckling piglets reached 100% (9, 10). The disease was shown to be caused by a highly pathogenic PEDV variant. The S genes of the classical CV777 strain and the new strain OH851 have the same insertions and deletions (S-INDEL strains), unlike those of the variant PEDV strains (11, 12).

The genome of PEDV is ~28 kb in length and contains seven open reading frames (ORFs), which encode four structural proteins and three non-structural proteins (13). S is the largest structural protein, and contains neutralizing antibody epitopes and a specific receptor-binding site for viral entry (14). At present, four antigenic epitopes have been characterized in the S protein, including the CO equivalent (COE) domain (amino acids 499–638), the epitopes SS2 (amino acids 748–755) and SS6 (amino acids 764–771), and epitope 2C10 (1368-GPRLQPY-1374) (15, 16). Because the S protein plays a vital role and the S gene is extensively mutated, it is often used as the target gene in the analysis of viral genetic variation. Based on whether the S gene contains the INDEL sequence or not, PEDV strains can be classified into genogroup II (GII) or genogroup I (GI), respectively. GI is further divided into two subgroups (GI-a, GI-b) according to INDEL sequence differences. At present, most isolates recovered in China belong to GII (17). A new mutation in the S gene of PEDV has recently been reported (18). Different GI-b strains have also been reported in different areas of China (19, 20). Studies have shown that PEDV strains of different genotypes can coexist, in one province in particular. These findings indicate that PEDV has continued to spread widely to most areas of China and has caused serious economic losses in the pig industry, reflecting the complex evolution of the virus. Therefore, extensive research into the evolutionary pathogenic mechanism of these strains in China is essential.

To control the spread of PEDV, a classical-CV777-derived vaccine has been widely used in many areas of China. However, it does not provide adequate protection against PEDV invasion (6, 21). In contrast, the wide-scale use of vaccines has increased the environmental stress upon the virus, causing PEDV to mutate to escape its host's immune defenses. To further and fully understand the prevalence and evolution of PEDV in southern China, diarrhea samples were collected from piglets in this study, and the variation of the S genes of the PEDV-positive samples were analyzed with sequence alignment and a phylogenetic tree.

## Materials and Methods

### Sample Collection

A total of 375 diarrheic samples from the small intestine tissues or feces were collected from suckling piglets on pig farms in eight provinces of China (Fujian, Guangdong, Guangxi, Guizhou, Jiangxi, Shandong, Hubei, Hu'nan, and Hainan) between June 2015 and October 2018. The piglets suffered severe watery diarrhea and dehydration. The diarrheic feces were resuspended in 1 mL of phosphate-buffered saline (PBS) in 1.5 mL Eppendorf tubes. After centrifugation at 10,000 × g for 5 min, 200 μL of each supernatant was transferred to a new tube for RNA extraction and virus isolation.

### RNA Extraction and Sequencing

The total RNA from the collected supernatants was extracted with TRIzol Reagent (TaKaRa), according to the manufacturer's instructions. The extracted RNA was subjected to reverse transcription (RT–PCR) with three pairs of newly designed primers to amplify and detect the PEDV S gene (Table 1). The three overlapping PCR products were identified with 1.5% agarose gel electrophoresis. The positive PCR products were sequenced by Sangon Biological Engineering Co. Ltd, and the entire sequence of the S gene was determined with the DNAStar software. The complete S gene sequences were submitted to GenBank, under the accession numbers shown in Table 2.

**Table 1:** Primers used for PEDV complete S gene amplification.

**Primer name**	**Nucleotide sequence, 5^**′**^-3^**′**^**	**Size(bp)**
PEDV S1-F	GGTAAGTTGCTAGTGCGTA	1,630
PEDV S1-R	CACAGAAAGAACTAAACCC	
PEDV S2-F	CTGCCATTCAGCGTATTCTTT	1,768
PEDV S2-R	CTGCGAGTTAACAACCTCTTGA	
PEDV S3-F	GTGCGCAGTATTACTCTGGT	1,559
PEDV S3-R	AAGAAGACGCTTTAAACAGTG	

**Table 2:** Information of S genes of 62 PEDV isolates.

**No**.	**Designation**	**Area**	**Region**	**Year**	**S (bp)**	**Accession no**
1	FJly15	Longyan	Fujian	2015	4161	MN368663
2	FJqz15	Quanzhou	Fujian	2015	4161	MN368664
3	FJzz15	Zhangzhou	Fujian	2015	4161	MN368665
4	GDgz15-1	Guangzhou	Guangdong	2015	4161	MN368666
5	GDgz15-2	Guangzhou	Guangdong	2015	4161	MN368667
6	GDhy15	Heyuan	Guangdong	2015	4161	MN368668
7	GDhz15	Huizhou	Guangdong	2015	4161	MN368669
8	GDjm15	Jiangmen	Guangdong	2015	4161	MN368670
9	GDmm15	Maoming	Guangdong	2015	4161	MN368671
10	GDsg15-1	Shaoguan	Guangdong	2015	4161	MN368672
11	GDsg15-2	Shaoguan	Guangdong	2015	4161	MN368673
12	GDsg15-3	Shaoguan	Guangdong	2015	4161	MN368674
13	GDzq15-1	Zhaoqing	Guangdong	2015	4161	MN368675
14	GDzq15-2	Zhaoqing	Guangdong	2015	4161	MN368676
15	GXnn15	Nanning	Guangxi	2015	4161	MN368678
16	GZgy15	Guiyang	Guizhou	2015	4161	MN368679
17	HBhg15	Huanggang	Hubei	2015	4161	MN368680
18	JXgz15	Ganzhou	Jiangxi	2015	4161	MN368681
19	JXyc15	Yichun	Jiangxi	2015	4161	MN368662
20	FJqz16	Quanzhou	Fujian	2016	4158	MN368683
21	GDfs16	Foshan	Guangdong	2016	4158	MN368684
22	GDhy16	Heyuan	Guangdong	2016	4161	MN368685
23	GDhz16	Huizhou	Guangdong	2016	4161	MN368686
24	GDjm16-1	Jiangmen	Guangdong	2016	4158	MN368687
25	GDjm16-2	Jiangmen	Guangdong	2016	4161	MN368688
26	GDjx16	Jiexi	Guangdong	2016	4158	MN368689
27	GDsg16-1	Shaoguan	Guangdong	2016	4158	MN368690
28	GDsg16-2	Shaoguan	Guangdong	2016	4158	MN368691
29	GDyj16	Ynagjiang	Guangdong	2016	4161	MN368692
30	GDgh16	Guanghui	Guangdong	2016	4158	MG983755
31	GDdg17	Dongguan	Guangdong	2016	4158	MN368693
32	FJfz17-1	Fuzhou	Fujian	2017	4161	MN368695
33	FJfz17-2	Fuzhou	Fujian	2017	4161	MN368696
34	FJqz17-1	Quanzhou	Fujian	2017	4161	MN368697
35	FJqz17-2	Quanzhou	Fujian	2017	4158	MN368698
36	GDhy17	Heyuan	Guangdong	2017	4158	MN368699
37	GDhz17	Huizhou	Guangdong	2017	4158	MN368700
38	GDjm17-1	Jiangmen	Guangdong	2017	4152	MN368701
39	GDjm17-2	Jiangmen	Guangdong	2017	4158	MN368702
40	GDjm17-3	Jiangmen	Guangdong	2017	4161	MN368703
41	GDmm17-1	Maoming	Guangdong	2017	4158	MN368704
42	GDmm17-2	Maoming	Guangdong	2017	4158	MN368705
43	GDsg17	Shaoguan	Guangdong	2017	4161	MN368706
44	HNcz17	Chenzhou	Hunan	2017	4161	MN368707
45	JXnc17	Nanchang	Jiangxi	2017	4158	MN368708
46	FJfz18-1	Fuzhou	Fujian	2018	4161	MN368710
47	FJfz18-2	Fuzhou	Fujian	2018	4158	MN368711
48	FJqz18	Quanzhou	Fujian	2018	4158	MN368712
49	GDhy18-1	Heyuan	Guangdong	2018	4158	MN368713
50	GDhy18-2	Heyuan	Guangdong	2018	4158	MN368714
51	GDhy18-3	Heyuan	Guangdong	2018	4158	MN368715
52	GDhz18	Huizhou	Guangdong	2018	4158	MN368716
53	GDjm18-1	Jiangmen	Guangdong	2018	4158	MN368717
54	GDjm18-2	Jiangmen	Guangdong	2018	4149	MN368718
55	GDmm18-1	Maoming	Guangdong	2018	4158	MN368719
56	GDmm18-2	Maoming	Guangdong	2018	4158	MN368720
57	GDsg18-1	Shaoguan	Guangdong	2018	4158	MN368721
58	GDsg18-2	Shaoguan	Guangdong	2018	4158	MN368722
59	GDst18	Shantou	Guangdong	2018	4158	MN368723
60	GDzj18-1	Zhanjiang	Guangdong	2018	4155	MN368724
61	GDzj18-2	Zhanjiang	Guangdong	2018	4161	MN368725
62	SDbz18	Binzhou	Shandong	2018	4158	MN368709

### S Gene Sequence Analysis

The complete genome sequences of reference strains available in GenBank were downloaded and used in a phylogenetic analysis (Table 3). A phylogenetic tree was constructed from all the S genes of the representative strains and isolates, using the neighbor joining method with 1,000 bootstrap replicates, with the Molecular Evolutionary Genetics Analysis (MEGA, version 6.0) software (22).

**Table 3:** Information of the representative strains.

**Virus strain**	**Countries**	**Year**	**Accession no**.	**Virus strain**	**Countries**	**Year**	**Accession no**.
CV777	Belgium	2001	AF353511	83P-5	Japan	2013	AB548618
JS-2004-2	China	2004	AY653204	OKN-1-JPN-2013	Japan	2013	LC063836
DX-S	China	2007	EU031893	CH-LXC-2014	China	2014	KT388418
LZC	China	2007	EF185992	PEDV-14	China	2014	KM609207
DR13/virulent	Korea	2007	DQ862099	CH-HNQX-3-14	China	2014	KR095279
JS2008	China	2008	KC109141	CH-HNYF-14	China	2014	KP890336
BJ-2011-1	China	2011	JN825712	CH-GD-22-2014	China	2014	KP870132
CH-JLCC-2011	China	2011	JQ638920	USA-Minnesota271-2014	USA	2014	KR265813
CH-S	China	2011	JN547228	MEX-124-2014	USA	2014	KJ645700
CH-FJND-1-2011	China	2011	JN543367	OH851	USA	2014	KJ399978
SM98	Korea	2011	GU937797	USA-Ohio126-2014	USA	2014	KJ645702
CH-GXNN-2012	China	2012	JX018179	AOM-2-JPN-2014	Japan	2014	LC063837
GD-A	China	2012	JX112709	AOM-3-JPN-2014	Japan	2014	LC063833
GD-B	China	2012	JX088695	KCH-2-JPN-2014	Japan	2014	LC063845
CH-SDDZ-2012	China	2012	KU133240	KPEDV-9	Korea	2014	KF898124
AH2012	China	2012	KC210145	KNU-1310	Korea	2014	KJ451045
JS-HZ2012	China	2012	KC210147	KNU-1401	Korea	2014	KJ451047
CH-ZJCX-1-2012	China	2012	KF840537	KNU-1406-1	Korea	2014	KM403155
CH9-FJ	China	2012	JQ979287	L00721-GER-2014	Germany	2014	LM645057
CV777/attenuated	China	2012	JN599150	FR-001-2014	France	2014	KR011756
CH7	China	2012	JQ239435	PEDV-WS	China	2015	KM609213
CH-HBXX2-11	China	2013	JX501319	CH-XBC-01-2015	China	2015	KR296677
CH-ZMDZY-11	China	2013	KC196276	CH-YGC-01-2015	China	2015	KR296678
CH-SBC-03-2013	China	2013	KC787542	CH-ZWBZa-01-2015	China	2015	KR296680
CH-YNKM-8-2013	China	2013	KF761675	CH-HNAY-2015	China	2015	KR809885
CH-JX-1-2013	China	2013	KF760557	CH-JPYC-02-2015	China	2015	JN547228
CH-HBQX-10	China	2013	JX501318	TW-Pingtung-63	China	2015	KP276250
USA-Indiana-17846-2013	USA	2013	KF452323	CBR2	Thailand	2015	KR610994
USA-Iowa-16465-2013	USA	2013	KF452322	HUA-PED47	Korea	2015	KP455314
USA-Minnesota90-2013	USA	2013	KJ645682	HUA-PED45	Korea	2015	KP455313
MN	USA	2013	KF468752	HUA-PED67	Korea	2015	KP455319
IA1	USA	2013	KF468753	15V010-BEL-2015	Belgium	2015	KR003452
IA2	USA	2013	KF468754	CH-HNCD-2016	China	2016	MF152600
NPL-PEDV-2013	USA	2013	KJ778615	HUA-14PED96	Korea	2016	KT941120
USA-Colorado-2013	USA	2013	KF272920	14JM-226	Japan	2018	KY619763
NK	Japan	2013	AB548623	14JM-126	Japan	2018	KY619740
MK	Japan	2013	AB548624	13JM-291	Japan	2018	KY619768

### Virus Isolation

Vero cells grown in a 24-well cell culture plate were infected with the previously prepared supernatants and maintained in Dulbecco's modified Eagle's medium (Thermo Scientific) containing 7 μg/mL trypsin without EDTA (Thermo Scientific). The cells were monitored daily for a cytopathic effect (CPE). When the CPE appeared in 70% of the cells, the cells were fixed with anhydrous ethanol. An immunofluorescence assay (IFA) was then performed with an anti-N protein monoclonal antibody (mAb; cat. # PEDV12-F, Alpha Diagnostic International Inc., USA) diluted 1:1,000 and an Alexa-Fluor®-488-conjugated Affinipure goat anti-mouse IgG(H+L) secondary antibody (SA00013-1; Proteintech, USA) diluted 1:400.

### Titer Determination for the Viral Proliferation Curve

Vero cells cultured in a 24-well cell culture plate were infected with PEDV at a multiplicity of infection (MOI) of 0.01. The cells and supernatants were collected at 12, 24, 36, 48, 60, 72, and 96 h post-infection (hpi). The cells were then frozen and thawed three times. After centrifugation at 10,000 × g for 5 min at 4°C, the supernatants were collected and the median tissue culture infective dose (TCID_50_) was determined with a microtitration infection assay.

### Piglet Challenge Experiment

To determine the virulence of the third-generation isolated strain GDgh16, six healthy 4-day-old colostrum-deprived suckling piglets were artificially fed bovine milk from birth. The colostrum-deprived piglets were randomly divided into two groups, with three piglets in each group. One group was challenged orally with 0.5 mL of PEDV at 10^5.0^ TCID_50_/mL. The other group received cell-culture medium. Duplicate samples of small intestine were collected in from all piglets, which had been euthanized at 48 h postchallenge. One of the duplicate samples was crushed in a grinder with 2 mL of PBS. The crushed intestine was then centrifuged at 10,000 × g for 10 min at 4°C. The supernatant was collected and their RNA extracted. The PEDV N gene copies in the small intestine were detected with real-time quantitative PCR (qPCR). RT–qPCR was performed with the PowerUp™ SYBR® Green Master Mix (A25742; Thermo Fisher) in a 20 μL reaction containing 1 ng of cDNA as the template, in a CFX96 thermal cycler, under the following cycling conditions: 50°C for 2 min; 95°C for 2 min; and 40 cycles of 95°C for 15 s, 60°C for 15 s, and 72°C for 60 s. The other sample was stained with anti-N protein mAb (diluted 1:1,000) for immunohistochemical (IHC) examination.

### Statistical Analysis

The numerical data are expressed as means ± standard deviations (SD), and all data were analyzed with the GraphPad Prism software (version 5.02 for Windows; GraphPad Software Inc.).

## Results

### PEDV Detection and Phylogenetic Analysis Based on the S Gene

As shown in Table 4, of the 375 feces and small -intestine samples tested between 2015 and 2018, 177 were positive for PEDV (47.20%). The positivity rates in 2015, 2016, 2017, and 2018 were 48.57% (34 positive samples and 70 test samples), 67.14%% (47 positive samples and 70 test samples), 53.33% (32 positive samples and 60 test samples), and 36.57% (64 positive samples and 175 test samples), respectively. The positive rate was highest in 2016 and lowest in 2018. A sequence alignment showed that these strains shared 92.9–100% nucleotide homology and 91–100% amino acid identity. They also shared 93.1–96.8% nucleotide homology and 91.5–96.8% amino acid identity with reference strain CV777, and 93.8–99% nucleotide homology and 92.5–98.9% amino acid identity with the reference strains isolated from China.

**Table 4:** The PEDV positive prevalence of different of tested strains in our study.

**Year**	**Province**	**Positive samples**	**Total samples**	**Positive prevalence**
2015	Fujian	3	7	42.86%
	Guangdong	26	58	44.83%
	Guangxi	1	1	100%
	Guizhou	1	1	100%
	Hubei	1	1	100%
	Jiangxi	2	2	100%
Total		34	70	48.57%
2016	Fujian	1	1	100%
	Guangdong	45	61	73.77%
	Hunan	1	1	100%
	Guangxi	0	3	0
	Jiangxi	0	4	0
Total		47	70	67.14%
2017	Fujian	4	7	57.14%
	Guangdong	18	34	52.94%
	Hu'nan	3	4	75%
	Guangxi	6	12	50%
	Jiangxi	1	3	33.33%
Total		32	60	53.33%
2018	Fujian	3	3	100%
	Guangdong	52	144	36.11%
	Guangxi	1	10	10%
	Jiangxi	3	10	30%
	Shandong	1	2	50%
	Hu'nan	0	2	0
	Hainan	4	4	100%
Total		64	175	36.57%
All total		177	375	47.20%

**Figure 1:**
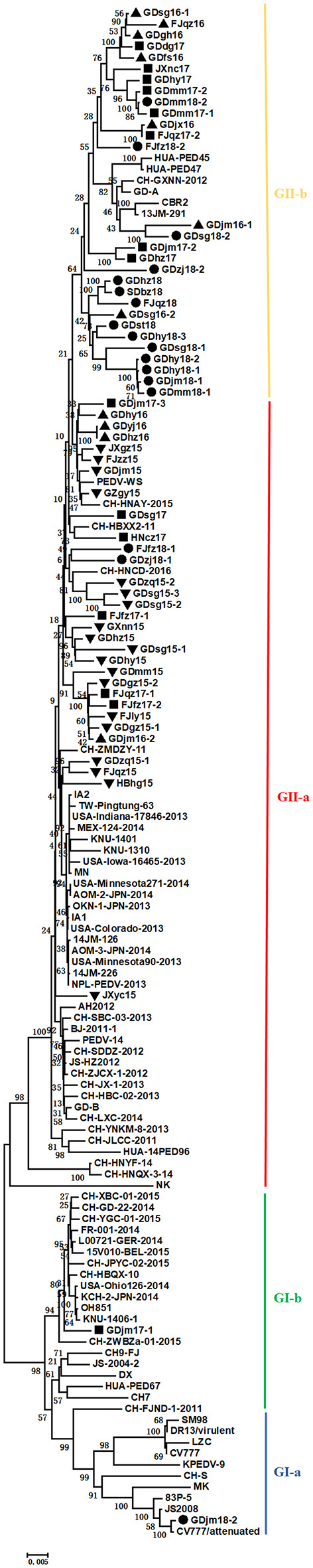
Phylogenetic tree based on the complete S genes of 62 Chinese PEDV strains identified in this study and other global reference strains. The tree was constructed with the neighbor-joining method in the MEGA V.6.0 program. The Chinese PEDV strains isolated in this study are marked with black triangles, black squares, or solid circles.

Sixty-two S genes from the test strains and representative strains downloaded from GenBank were analyzed with a phylogenetic tree. As shown in Figure 1, the phylogenetic analysis divided these strains into two groups, GI and GII, based on whether the S gene contained the S-INDEL (23). GI included the classical strains (CV777 and SM98) and some isolates from China, the USA, and Japan collected after 2010. Therefore, GI was further divided into three subgroups: GI-a, GI-b. GI-a contained classical S-INDEL strains. GI-b contained a new S-INDEL strain. GII contained non-S-INDEL strains and was also divided into two subgroups, GII-a and GII-b, which consisted of a number of extremely virulent strains from all over the world, isolated since 2010. The strains isolated in the present study belonged to GI-a, GI-b, GII-a, and GII-b. GDjm18-2, was categorized as subtype GI-a, which also included the classical vaccine strains CV777-attenuated and JS2008. GDjm17-1 was categorized in GI-b cluster. The other strains identified in the present study formed eight clusters. Of these strains, 25 isolates from Guangdong, three isolates from Fujian, and one isolate from Jiangxi formed three clusters and belonged to GII-b, with strong similarity to GD-A and CH-GXNN-2012. The other 34 isolates formed five clusters and belonged to GII-a. Among these 34 strains, JXyc15 was closely related to the C4 cluster (North American strains), whereas the other strains showed closer identity to CH-ZMDZ-11, CH-HNAY-2015, and CH-HNCDE-2016L. As shown in Table 5, all the strains isolated in 2015 belonged to GII-a (100%). In 2016 and 2017, 46.15% and 43.75% of the isolated strains belonged to GII-a, respectively. Compared with GII-a, the rate slightly increased, and in 2016 and 2017, 53.84 and 50% of the isolated strains belonged to GII-b, respectively. However, in 2018, 72.22% of the isolated strains belonged to GII-b, which was much higher than the proportion that belonged to GII-a in 2017 (22.22%). The comparison result show: Variation of PEDV S gene is continuously occurring and GII-b strains may be the dominant strains in China in the future.

**Table 5:** The PEDV positive prevalence of different groups of tested strains in our study.

**Group**	**2015**	**2016**	**2017**	**2018**
GI-a	0	0	0	5.56%
GI-b	0	0	6.25%	0
GII-a	100%	46.15%	43.75%	22.22%
GII-b	0	53.84%	50%	72.22%

### Amino Acid Sequence Analysis of Neutralizing Epitopes in the S Protein

**Figure 2:**
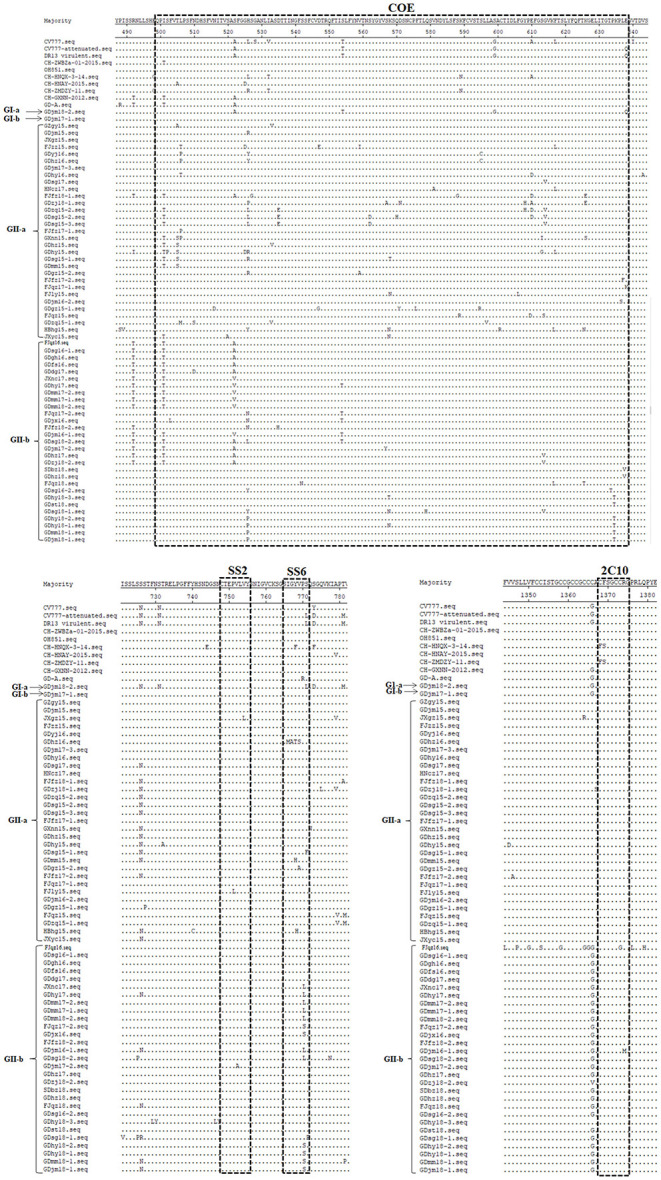
Amino acid sequence analysis of the neutralizing epitopes of the S protein. Amino acid sequence alignment of the neutralizing epitopes of the S proteins of the isolated strains and reference strains, constructed with the Clustal W method.

Neutralizing antibodies play an important role in the prevention and control of viral infections. Therefore, it is important to identify and analyze the amino acid sequences of the neutralizing epitopes in viral proteins. To analyze the genetic characteristics of the South China PEDV strains, the deduced amino acid sequences of the S proteins detected in our study were aligned and compared with those of representative PEDV strains, including strains from GI-a (CV777 and DR13 virulent), GI-b (OH851 and CH-ZWZBa-01-2015), GII-a (CH-HNQX-3-14, CH-HNAY-2015, CH-ZMDZY-11), and GII-b (CH-GXNN-2012, CD-A). As shown in Figure 2, compared with strain CV777, the GI-a strain GDjm18-2 had three amino acid substitutions in the COE domain and one amino acid substitution in epitope SS6. The GII-a strains had amino acid substitutions at 35 positions in the COE domain, at two positions in epitope SS2, and at five positions in epitope SS6. Many new amino acid substitutions were detected in the COE regions of the GII-a strains, at positions 502 (S→ P), 507 (P→ M), 510 (N→ S), 516 (N→ D), 522 (S→ A), 527 (S→ G), 533 (A→ V), 535 (D→ E), 547 (D→ E), 559 (V→ I or A), 562 (S→ D), 567 (S→ A), 568 (K→ T or N), 570 (Q→ H), 571 (D→ N or Y), 575 (P→ L), 580 (S→ A), 588 (S→ G), 594 (T→ R or C), 608 (Y→ H), 613 (S→ I or G), 614 (G→ V), 626 (K→ E or S), and 637 (L→ F or S). Epitope 2C10 was conserved in all GII-a strains. Among the GII-a strains, GDhz16 had four continuous amino acid mutations in epitope SS6, which differed from the epitope sequence in the other strains and the reference strains. Compared with strain CV777, the GII-b strains had amino acid substitutions at 17 positions in the COE domain, and amino acid substitutions at one position in three epitopes (SS2, SS6, and 2C10). As well as the common amino acid mutations that were similar to those in the GII-b reference strains, there were novel amino acid substitutions at eight positions in the COE region: 504 (V→ L), 510 (N→ D), 535 (D→ H), 542 (S→ H), 567 (S→ Y), 614 (G→ V), 626 (K→ T), and 637 (L→ V). These amino acid sequences demonstrate that the neutralizing epitopes of the PEDV S protein are constantly mutating. This phenomenon increases the difficulty of preventing and controlling PEDV infections because existing vaccines cannot effectively protect against PEDV. However, these finding may facilitate the development of effective novel vaccines in the future.

### Numbers of Mutated Amino Acid in Different Domains of the S Protein

**Figure 3:**
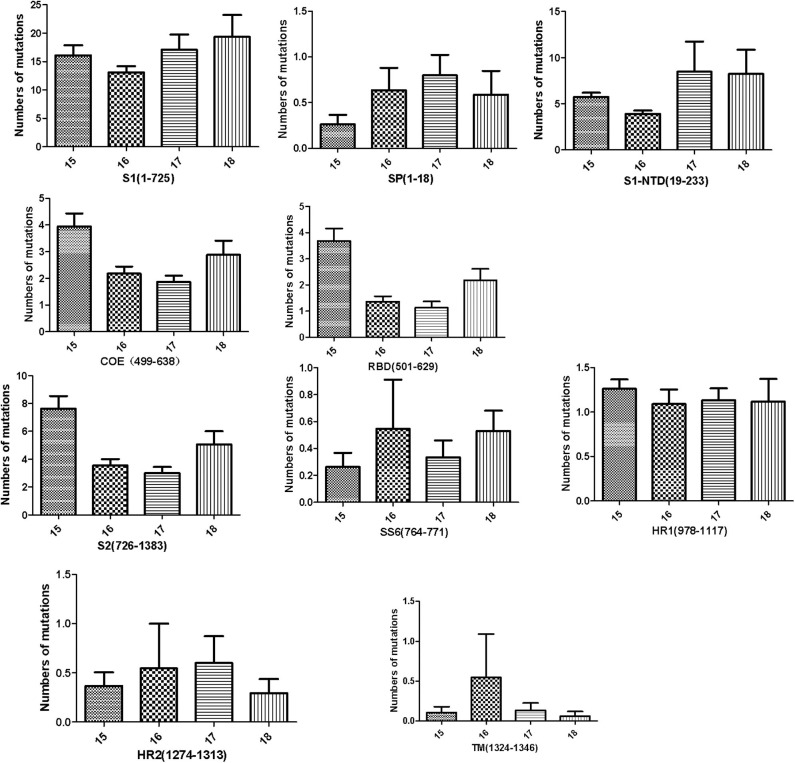
Numbers of amino acid mutations in different domains of the S protein, including the S1 subunit (residues 1–725), S2 subunit (residues 726–1383), signal peptide (SP, residues 1–18), N-terminal domain of S1 (S1-NTD, residues 19–233), the neutralizing epitopes (COE, residues 499–638; SS2, residues 748–755; SS6, residues 764–771; 2C10, residues 1368–1374), two heptad repeat regions (HR1, residues 978–1117, and HR2, residues 1274–1313), and the transmembrane domain (TM, residues 1324–1346).

To further analyze the amino acid mutations in the different domains of the S protein in these isolates, the different domains of the S protein were aligned with those of CV777, and the average number of amino acid mutations present in each year was calculated. The S protein can be divided into the S1 protein and the S2 protein. The S1 protein contains four domains: SP (amino acids 1–18), S1-NTD (amino acids 19–233), COE and RBD (amino acids 501–629), whereas the S2 protein contains five domains: SS6 (amino acids 764–771), HR1 (amino acids 978–1117), HR2 (amino acids 1274–1313), TM (amino acids 1324–1346), and 2C10 (amino acids 1368–1374). Previous data have indicated that 2C10 is conserved, so we did not analyze the 2C10 domain. As shown in Figure 3, in these strains, the S1 sequence had more amino acid mutations than the S2 sequence. From 2015 to 2018, the number of mutated amino acids in S1 remained at a high level, whereas that in S2 decreased. Furthermore, the numbers of mutated amino acids in SP (amino acids 1–18) and S1-NTD (amino acids 19–233) increased slightly, whereas the numbers of mutated amino acids in the COE and RBD domains decreased. SS6, HR1, HR2, and TM in the S2 protein did not change obviously from 2015 to 2018.

### Pathogenicity of GDgh16

**Figure 4:**
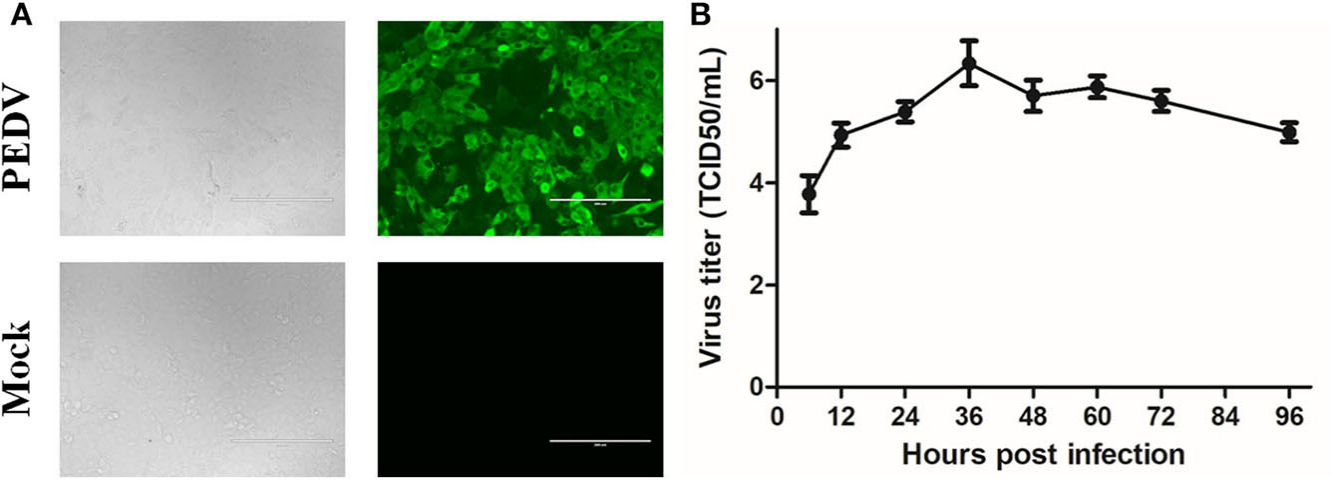
Detection and proliferation curve of PEDV strain GDgh16. **(A)** Identification of GDgh16 in Vero cells. CPE of GDgh16 was observed at 24 hpi under white light and was tested with IFA using a monoclonal antibody directed against the PEDV N protein. **(B)** Proliferation curve of PEDV strain GDgh16. Vero cells were infected with GDgh16 at a multiplicity of infection of 0.01. Cells and culture solution were collected at 6, 12, 24, 36, 48, 60, and 72 hpi, frozen, thawed, and centrifuged. The supernatant was collected and TCID_50_ determined.

**Figure 5:**
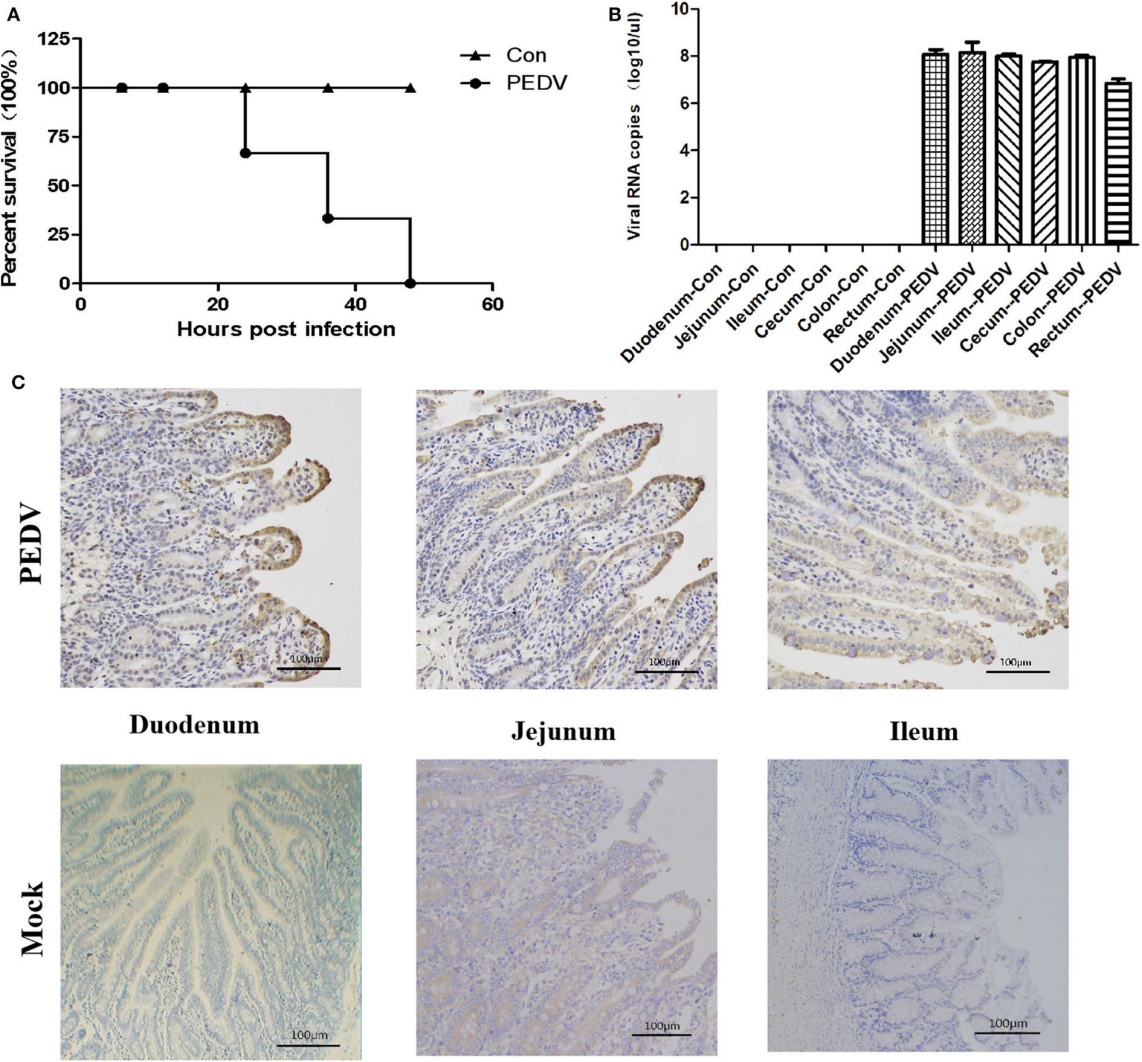
Pathogenicity analysis of GDgh16. **(A)** Survival rate of piglets in each group. **(B)** Quantification of the viral loads in different parts of the intestine. Different parts of the intestine were isolated and the viral load was quantified with TaqMan real-time RT–PCR targeting the PEDV N gene. **(C)** Immunohistochemical analysis of intestines. Duodenum, jejunum, and ileum tissues from each group were stained with monoclonal antibody directed against PEDV N protein (diluted 1:100).

Because the samples of GDgh16 came from a scale pig farm that had experienced high mortality, and it displayed a high viral titer in Vero cells, we investigated its pathogenicity *in vivo*. As shown in Figure 4A, PEDV-infected cells showed a characteristic green color, indicating that PEDV was isolated successfully. The viral proliferation curve indicated that the titer of strain GDgh16 increased to 10^6.33^ TCID_50_/mL at 36 hpi but decreased to 10^4.99^ TCID_50_/mL at 96 hpi (Figure 4B). Six piglets were divided into two groups; one group was challenged orally with GDgh16 and the other group was inoculated with cell culture medium. All the challenged piglets showed classical clinical signs, including vomiting, watery diarrhea, and dehydration, at 16 hpi. The challenged piglets began to die at 24 hpi, and all had died by 48 hpi (Figure 5A). The control pigs remained healthy, with no detectable PEDV shedding. The control piglets were euthanized and necropsy was performed on all the piglets. The viral copy numbers in different parts of the intestine were determined with RT–qPCR. The duodenum, jejunum, ileum, cecum, and colon had higher viral copy numbers than the rectum (Figure 5B). The duodenums, jejunums, and ileums of the piglets were subjected to an IHC assay. As shown in Figure 5C, the tissues from the piglets in the challenged group showed remarkable levels of viral antigens compared to those in the control group. The results of GDgh16 challenge test indicate that the variant strains are a large threat to the pig industry and that the control of PEDV spread has become a critical issue.

## Discussion

PEDV has become an important diarrhea virus, causing extensive damage to pig farms worldwide. Because there is no effective vaccine against the emerging prevalent strains in China, the variant PEDV strains occur frequently on many farms in different areas (24). Because there are extensive viral variants and the protection afforded by commercial vaccines is limited, it is necessary to fully understand the genetic variations and epidemiology of PEDV to facilitate the development of next-generation vaccines.

In the present study, the genetic variations in PEDV in parts of China in 2015–2018 were analyzed.

The S gene encodes the largest structural protein of PEDV and stimulates the host body to produce neutralizing antibodies against the virus. Because its variants are extensive, the S gene is commonly used as the target gene in studies of the genomic characteristics of PEDV (25). A phylogenetic analysis showed that strains from four subgroups of PEDV were present from 2015 to 2018, and that GII-a and GII-b were the two most prevalent subgroups in China at that time. From 2015 to 2018, eight strains belonging to four subgroups (GI-a, GI-b, GII-a, and GII-b) were epidemic in Jiangmen (Guangdong), which suggests that PEDV had mutated widely and the PEDV epidemic was becoming more complex. These results are consistent with those of Wen et al. (26). In 2015, all the isolated strains belonged to GII-a, whereas in 2018, 72.22% of strains belonged to GII-b, and only 22.22% of strains belonged to GII-a. Interestingly, unlike GII-a, which includes strains from other countries, such as America, South Korea, and Japan, the GII-b subgroup only contains Chinese-isolated strains. Combined with previous studies, these results suggest that GII-b strains may be the dominant strains in China in the future (27, 28).

The S protein is highly variable, and many studies have shown that amino acid changes in the S protein can affect the virulence and pathogenicity of PEDV. Our study has shown that the numbers of amino acid mutations in the SP1 and S1-NTD domains of PEDV increased in 2017 and 2018. It had been suggested that S1-NTD is a vital domain related to viral virulence (29, 30) and that conformational changes in S1-NTD are related to the high pathogenicity of PEDV strain FJzz1 (18, 27). Increasing numbers of more-virulent PEDV strains have recently emerged (18, 27, 31). Whether the mutations identified in this study alter the major conformation and thus the pathogenicity of these strains will be investigated further in the future. Our data show that the PEDV positivity rate in the provinces tested increased from 2015 to 2016, but decreased from 2016 to 2018, which might be attributable to improvements in disease prevention and control strategies. Many pig farms use the “feed-back” mode to ensure sow immunity to PEDV and to protect piglets against PEDV infection. This is an effective measure to prevent PED, but there is also a risk of virus dispersal, which is responsible for the many GI-b strains reported to date (19, 20, 32–34).

Four neutralizing epitopes of the PEDV S protein have been determined: the COE domain (499–638), epitope SS2 (748–755), epitope SS6 (764–771), and epitope 2C10 (1368–1374) (15, 16). In the present study, we detected amino acid changes at 35 positions in the COE domain. Moreover, one strain, GDhz16, had four continuous amino acid mutations in epitope SS6. Epitopes SS2 and 2C10 also contained amino acid substitutions. The antigenicity, pathogenicity, and neutralization properties of isolated strains are altered by such mutations, especially some insertions and deletions in the S protein (35, 36). Therefore, the vaccine derived from prototype strain CV777 protects against the disease induced by classical strains but not the disease caused by variant strains (24, 37). Whether these amino acid changes affect the antigenicity and neutralization properties of the four neutralizing epitopes warrants investigation in future studies.

Based on previous epidemiological and clinical observations of field strains since 2010, the emerging GII strains are highly pathogenic (38). To investigate the pathogenicity of the isolated variant strains, three piglets were infected orally with GDgh16. The piglets in the infected group began to show clinical signs of diarrhea at 12 h, and developed the typical symptoms of PED at 16 h. Morbidity reached 100%. The piglets began to die at 24 hpi, and all had died by 48 hpi. Moreover, their small intestines contained high viral copies and many viral antigens, indicating that GDgh16 was a highly pathogenic strain. Other researchers have demonstrated that different types of pigs infected with variant PEDV strains shared consistent outcomes (39–42). These results indicate that the variant strains are a large threat to the pig industry, and that the control of PEDV spread has become a critical issue.

In conclusion, the PEDV strains circulating in parts of China between 2015 and 2018 clustered into four subgroups: GI-a, GI-b, GII-a, and GII-b. The GII-b strains became dominant in 2018. Compared with previous strains, these strains displayed multiple variations in the SP and S1-NTD domains and the neutralizing epitopes of the S protein. We successfully isolated and identified a new virulent GII-b strain, GDgh16, which is well-adapted to Vero cells and causes a high mortality rate in piglets. Our study provides insight into the genetic characteristics of the prevalent PEDV strains in parts of China, and suggests that the development of effective novel vaccines is both necessary and urgent.

## Data Availability Statement

The datasets presented in this study can be found in online repositories. The names of the repository/repositories and accession number(s) can be found in the article/supplementary material.

## Ethics Statement

The animal study was reviewed and approved by The National Engineering Center for Swine Breeding Industry (NECSBI 2015-16).

## Author Contributions

LY, YL, JD, and CS conceived and designed the experiments. LY, YL, SW, LZ, PL, and LW performed the experiments. LY, JD, and CS analyzed the data and wrote the paper. All authors read and approved the final manuscript.

## Conflict of Interest

The authors declare that the research was conducted in the absence of any commercial or financial relationships that could be construed as a potential conflict of interest.

## References

[1] Pensaert MB de Bouck P. A new coronavirus-like particle associated with diarrhea in swine. Arch Virol. (1978) 58:243–7. 10.1007/bf01317606 83132 PMC7086830

[2] Boniotti MB Papetti A Lavazza A Alborali G Sozzi E Chiapponi C. Porcine epidemic diarrhea virus and discovery of a recombinant swine enteric coronavirus, Italy. Emerg Infect Dis. (2016) 22:83–7. 10.3201/eid2201.150544 26689738 PMC4696687

[3] Kim YK Cho YY An BH Lim SI Lim JA Cho IS . Molecular characterization of the spike and ORF3 genes of porcine epidemic diarrhea virus in the Philippines. Arch Virol. (2016) 161:1323–8. 10.1007/s00705-016-2758-2 26801789

[4] Pasick J Berhane Y Ojkic D Maxie G Embury-Hyatt C Swekla K. Investigation into the role of potentially contaminated feed as a source of the first-detected outbreaks of porcine epidemic diarrhea in Canada. Transbound Emerg Dis. (2014) 61:397–410. 10.1111/tbed.12269 25098383 PMC4282400

[5] Steinrigl A Fernandez SR Stoiber F Pikalo J Sattler T Schmoll F. First detection, clinical presentation and phylogenetic characterization of Porcine epidemic diarrhea virus in Austria. Bmc Vet Res. (2015) 11:310. 10.1186/s12917-015-0624-1 26714453 PMC4696200

[6] Sun RQ Cai RJ Chen YQ Liang PS Chen DK Song CX. Outbreak of porcine epidemic diarrhea in suckling piglets, China. Emerg Infect Dis. (2012) 18:161–3. 10.3201/eid1801.111259 22261231 PMC3381683

[7] Tian Y Yu Z Cheng K Liu Y Huang J Xin Y. Molecular characterization and phylogenetic analysis of new variants of the porcine epidemic diarrhea virus in Gansu, China in 2012. Viruses. (2013) 5:1991–2004. 10.3390/v5081991 23955500 PMC3761238

[8] Sung MH Deng MC Chung YH Huang YL Chang CY Lan YC. Evolutionary characterization of the emerging porcine epidemic diarrhea virus worldwide and 2014 epidemic in Taiwan. Infect Genet Evol. (2015) 36:108–15. 10.1016/j.meegid.2015.09.011 26375730 PMC7106162

[9] Stevenson GW Hoang H Schwartz KJ Burrough ER Sun D Madson D. Emergence of Porcine epidemic diarrhea virus in the United States: clinical signs, lesions, and viral genomic sequences. J Vet Diagn Invest. (2013) 25:649–54. 10.1177/1040638713501675 23963154

[10] Jung K Saif LJ. Porcine epidemic diarrhea virus infection: etiology, epidemiology, pathogenesis and immunoprophylaxis. Vet J. (2015) 204:134–43. 10.1016/j.tvjl.2015.02.017 25841898 PMC7110711

[11] Mesquita JR Hakze-van DHR Almeida A Lourenco M van der Poel WH Nascimento MS. Outbreak of porcine epidemic diarrhea virus in Portugal, 2015. Transbound Emerg Dis. (2015) 62:586–8. 10.1111/tbed.12409 26344708 PMC7169791

[12] Wang L Byrum B Zhang Y. New variant of porcine epidemic diarrhea virus, United States, 2014. Emerg Infect Dis. (2014) 20:917–9. 10.3201/eid2005.140195 24750580 PMC4012824

[13] Jarvis MC Lam HC Zhang Y Wang L Hesse RA Hause BM. Genomic and evolutionary inferences between American and global strains of porcine epidemic diarrhea virus. Prev Vet Med. (2016) 123:175–84. 10.1016/j.prevetmed.2015.10.020 26611651 PMC7114344

[14] Wang D Ge X Chen D Li J Cai Y Deng J. The S gene is necessary but not sufficient for the virulence of porcine epidemic diarrhea virus novel variant strain BJ2011C. J Virol. (2018) 92:e00603-18. 10.1128/JVI.00603-18 29695430 PMC6002738

[15] Chang SH Bae JL Kang TJ Kim J Chung GH Lim CW. Identification of the epitope region capable of inducing neutralizing antibodies against the porcine epidemic diarrhea virus. Mol Cells. (2002) 14:295–9. 12442904

[16] Sun D Feng L Shi H Chen J Cui X Chen H. Identification of two novel B cell epitopes on porcine epidemic diarrhea virus spike protein. Vet Microbiol. (2008) 131:73–81. 10.1016/j.vetmic.2008.02.022 18400422 PMC7117171

[17] Zhang Q Liu X Fang Y Zhou P Wang Y Zhang Y. Detection and phylogenetic analyses of spike genes in porcine epidemic diarrhea virus strains circulating in China in 2016-2017. Virol J. (2017) 14:194. 10.1186/s12985-017-0860-z 29017599 PMC5634871

[18] Liu X Zhang Q Zhang L Zhou P Yang J Fang Y. A newly isolated Chinese virulent genotype GIIb porcine epidemic diarrhea virus strain: biological characteristics, pathogenicity and immune protective effects as an inactivated vaccine candidate. Virus Res. (2019) 259:18–27. 10.1016/j.virusres.2018.10.012 30342075 PMC7111334

[19] Wang P Zhu J Liu X Guo J Gu X Ruan W. Isolation and recombinant analysis of variants of porcine epidemic diarrhea virus strains from Beijing, China. Virus Dis. (2019) 30:294–301. 10.1007/s13337-019-00513-w 31179369 PMC6531531

[20] Chen N Li S Zhou R Zhu M He S Ye M. Two novel porcine epidemic diarrhea virus (PEDV) recombinants from a natural recombinant and distinct subtypes of PEDV variants. Virus Res. (2017) 242:90–5. 10.1016/j.virusres.2017.09.013 28947336

[21] Wang X Niu B Yan H Gao D Yang X Chen L. Genetic properties of endemic Chinese porcine epidemic diarrhea virus strains isolated since 2010. Arch Virol. (2013) 158:2487–94. 10.1007/s00705-013-1767-7 23797760 PMC7087078

[22] Kumar S Stecher G Li M Knyaz C Tamura K. MEGA X: molecular evolutionary genetics analysis across computing platforms. Mol Biol Evol. (2018) 35:1547–9. 10.1093/molbev/msy096 29722887 PMC5967553

[23] Wang D Fang L Xiao S. Porcine epidemic diarrhea in China. Virus Res. (2016) 226:7–13. 10.1016/j.virusres.2016.05.026 27261169 PMC7114554

[24] Li W Li H Liu Y Pan Y Deng F Song Y. New variants of porcine epidemic diarrhea virus, China, 2011. Emerg Infect Dis. (2012) 18:1350–3. 10.3201/eid1808.120002 22840964 PMC3414035

[25] Chen J Liu X Shi D Shi H Zhang X Li C. Detection and molecular diversity of spike gene of porcine epidemic diarrhea virus in China. Viruses. (2013) 5:2601–13. 10.3390/v5102601 24153062 PMC3814607

[26] Wen Z Li J Zhang Y Zhou Q Gong L Xue C. Genetic epidemiology of porcine epidemic diarrhoea virus circulating in China in 2012-2017 based on spike gene. Transbound Emerg Dis. (2018) 65:883–9. 10.1111/tbed.12825 29388343 PMC7169843

[27] Chen P Wang K Hou Y Li H Li X Yu L. Genetic evolution analysis and pathogenicity assessment of porcine epidemic diarrhea virus strains circulating in part of China during 2011-2017. Infect Genet Evol. (2019) 69:153–65. 10.1016/j.meegid.2019.01.022 30677534 PMC7106134

[28] Yu J Chai X Cheng Y Xing G Liao A Du L. Molecular characteristics of the spike gene of porcine epidemic diarrhoea virus strains in Eastern China in 2016. Virus Res. (2018) 247:47–54. 10.1016/j.virusres.2018.01.013 29412159

[29] Hou Y Lin CM Yokoyama M Yount BL Marthaler D Douglas AL. Deletion of a 197-amino-acid region in the N-terminal domain of spike protein attenuates porcine epidemic diarrhea virus in piglets. J Virol. (2017) 91:e00227-17. 10.1128/JVI.00227-17 28490591 PMC5487580

[30] Su Y Hou Y Prarat M Zhang Y Wang Q. New variants of porcine epidemic diarrhea virus with large deletions in the spike protein, identified in the United States, 2016-2017. Arch Virol. (2018) 163:2485–9. 10.1007/s00705-018-3874-y 29789941 PMC7087112

[31] Zhang L Liu X Zhang Q Zhou P Fang Y Dong Z. Biological characterization and pathogenicity of a newly isolated Chinese highly virulent genotype GIIa porcine epidemic diarrhea virus strain. Arch Virol. (2019) 164:1287–95. 10.1007/s00705-019-04167-3 30859476 PMC7086859

[32] Li B Liu H He K Guo R Ni Y Du L. Complete genome sequence of a recombinant porcine epidemic diarrhea virus strain from eastern china. Genome Announc. (2013) 1:e10513. 10.1128/genomeA.00105-13 23599287 PMC3630398

[33] Li K Song D Zhang F Gong W Guo N Li A. Complete genome sequence of a recombinant porcine epidemic diarrhea virus strain, CH/JXJA/2017, isolated in jiangxi, China, in 2017. Genome Announc. (2018) 6:e01590-17. 10.1128/genomeA.01590-17 29439052 PMC5805890

[34] Li R Qiao S Yang Y Guo J Xie S Zhou E. Genome sequencing and analysis of a novel recombinant porcine epidemic diarrhea virus strain from Henan, China. Virus Genes. (2016) 52:91–8. 10.1007/s11262-015-1254-1 26743534 PMC7089116

[35] Park S Kim S Song D Park B. Novel porcine epidemic diarrhea virus variant with large genomic deletion, South Korea. Emerg Infect Dis. (2014) 20:2089–92. 10.3201/eid2012.131642 25424875 PMC4257805

[36] Zhang X Pan Y Wang D Tian X Song Y Cao Y. Identification and pathogenicity of a variant porcine epidemic diarrhea virus field strain with reduced virulence. Virol J. (2015) 12:88. 10.1186/s12985-015-0314-4 26063495 PMC4504071

[37] Puranaveja S Poolperm P Lertwatcharasarakul P Kesdaengsakonwut S Boonsoongnern A Urairong K. Chinese-like strain of porcine epidemic diarrhea virus, Thailand. Emerg Infect Dis. (2009) 15:1112–5. 10.3201/eid1507.081256 19624933 PMC2744260

[38] Lin CM Saif LJ Marthaler D Wang Q. Evolution, antigenicity and pathogenicity of global porcine epidemic diarrhea virus strains. Virus Res. (2016) 226:20–39. 10.1016/j.virusres.2016.05.023 27288724 PMC7111424

[39] Madson DM Arruda PH Magstadt DR Burrough ER Hoang H Sun D. Characterization of porcine epidemic diarrhea virus isolate US/Iowa/18984/2013 infection in 1-day-old cesarean-derived colostrum-deprived piglets. Vet Pathol. (2016) 53:44–52. 10.1177/0300985815591080 26113613

[40] Chen Q Gauger PC Stafne MR Thomas JT Madson DM Huang H. Pathogenesis comparison between the United States porcine epidemic diarrhoea virus prototype and S-INDEL-variant strains in conventional neonatal piglets. J Gen Virol. (2016) 97:1107–21. 10.1099/jgv.0.000419 26841768

[41] Lin CM Annamalai T Liu X Gao X Lu Z El-Tholoth M. Experimental infection of a US spike-insertion deletion porcine epidemic diarrhea virus in conventional nursing piglets and cross-protection to the original US PEDV infection. Vet Res. (2015) 46:134. 10.1186/s13567-015-0278-9 26589292 PMC4654902

[42] Thomas JT Chen Q Gauger PC Gimenez-Lirola LG Sinha A Harmon KM. Effect of porcine epidemic diarrhea virus infectious doses on infection outcomes in naive conventional neonatal and weaned pigs. PLoS ONE. (2015) 10:e139266. 10.1371/journal.pone.0139266 26441071 PMC4594914

[43] Yu L Liu Y Wang S Zhang L Liang P Wang L Molecular characteristics and pathogenicity of porcine epidemic diarrhea virus in some areas of China from 2015 to 2018. Res Square. (2020). 10.21203/rs.3.rs-41418/v1 PMC7793843 33426027

